# Invasive floating macrophytes reduce greenhouse gas emissions from a small tropical lake

**DOI:** 10.1038/srep20424

**Published:** 2016-02-05

**Authors:** K. Attermeyer, S. Flury, R. Jayakumar, P. Fiener, K. Steger, V. Arya, F. Wilken, R. van Geldern, K. Premke

**Affiliations:** 1 Leibniz-Institute of Freshwater Ecology and Inland Fisheries, Chemical Analytics and Biogeochemistry, Müggelseedamm 310, 12587 Berlin, Germany; 2 University of Geneva, Faculty of Science, Boulevard Carl-Vogt 66, 1211 Geneva, Switzerland; 3 Indo-German Centre for Sustainability (IGCS), Indian Institute of Technology Madras (IITM), Chennai 600 036, India; 4 Indian Institute of Technology Madras (IITM), Environmental and Water Resources Engineering Division, Department of Civil Engineering, Chennai 600 036, India; 5 University of Augsburg, Department of Geography, Alter Postweg 118, 86159 Augsburg, Germany; 6 Brandenburg University of Technology (BTU), Chair of Soil Protection and Recultivation, Konrad-Wachsmann-Allee 6, 03013 Cottbus, Germany; 7 Friedrich-Alexander University Erlangen-Nuremberg (FAU), GeoZentrum Nordbayern, Schlossgarten 5, 91054 Erlangen, Germany; 8 Leibniz Centre for Agricultural Landscape Research (ZALF), Institute for Landscape Biogeochemistry, Eberswalder Straße 84, 15374 Müncheberg, Germany

## Abstract

Floating macrophytes, including water hyacinth (*Eichhornia crassipes*), are dominant invasive organisms in tropical aquatic systems, and they may play an important role in modifying the gas exchange between water and the atmosphere. However, these systems are underrepresented in global datasets of greenhouse gas (GHG) emissions. This study investigated the carbon (C) turnover and GHG emissions from a small (0.6 km^2^) water-harvesting lake in South India and analysed the effect of floating macrophytes on these emissions. We measured carbon dioxide (CO_2_) and methane (CH_4_) emissions with gas chambers in the field as well as water C mineralization rates and physicochemical variables in both the open water and in water within stands of water hyacinths. The CO_2_ and CH_4_ emissions from areas covered by water hyacinths were reduced by 57% compared with that of open water. However, the C mineralization rates were not significantly different in the water between the two areas. We conclude that the increased invasion of water hyacinths and other floating macrophytes has the potential to change GHG emissions, a process that might be relevant in regional C budgets.

Tropical and subtropical regions are encountering increasing abundances of invasive floating macrophyte species^1^,^2^, and such free-floating plant communities often outcompete submerged macrophytes or phytoplankton and represent an alternative stable state in shallow lakes^3^,^4^. However, aquatic macrophytes perform important ecosystem functions, particularly in shallow ecosystems, where they may act as engineer species, changing the structure of the ecosystems that they colonize^5^. These plant communities are sources of organic matter and sinks for nutrients, and they can also act as important regulators of gas exchanges between the sediment, the water and the atmosphere^6^.

Most of India’s water bodies are small (<1 km^2^) water-harvesting ponds and lakes that are often characterized by high nutrient inputs and substantial floating macrophyte coverage^7^. A common floating macrophyte in India is the invasive water hyacinth (*Eichhornia crassipes*), which is native to lowlands of South America^8^. This plant has been present in India since 1890^9^, and its prevalence has substantially increased since 1998^9^. Because of its rapid growth rate, which can double the biomass within five days, and its ability to successfully compete with other aquatic plants, water hyacinths now cover more than 2,000 km^2^ of the freshwater bodies in India, which corresponds to 10% - 15% of the total area covered by aquatic vegetation^7^,^10^.

According to Scheffer and co-authors^3^ the invasion of free-floating plants is among the most important threats to the functioning and biodiversity of aquatic ecosystems. These plants negatively affect fishing operations, obstruct or even prevent water traffic, impede irrigation and hamper hydropower generation^11^. Furthermore, water hyacinth is known to change the physicochemical characteristics of water (e.g., the pH, alkalinity, dissolved oxygen (O_2_) concentration and dissolved carbon dioxide (CO_2_) concentration)^12^. For example, O_2_ in the water can be diminished by emergent macrophytes, which limit pelagic and benthic photosynthesis through shading^13^ and prohibit gas exchange and thus re-oxygenation from the atmosphere. Compared with the O_2_ produced by submersed aquatic plants and phytoplankton, O_2_ that is photosynthetically produced by emerged macrophytes is directly emitted into the atmosphere and does not contribute to aquatic O_2_ concentrations^14^. C turnover underneath the water hyacinths can be further fuelled by root respiration and microbial activity in the water and sediments because of dissolved organic matter from root exudates and decaying plant litter^15^,^16^,^17^, which eventually increase CO_2_ and CH_4_ concentrations below these floating plants. Therefore, water hyacinths have considerable ecological impacts, which may confer unwanted economic effects^18^. However, most studies of water hyacinths have examined their effects on water quality and their dispersal spread or phytoremediation^19^ (and references therein). The link between the invasion of water hyacinths and the emission of climate-relevant gases (CO_2_ and CH_4_) has not yet been explored.

Most freshwater systems are net greenhouse gas (GHG) emitters^20^,^21^. CO_2_ and CH_4_ are among the major gases impacting the atmospheric heat budget and contributing to global climate change. Consequently, investigations of GHG emissions and their influencing factors are of major importance for understanding current and predicting future climate conditions. Most GHG research in inland waters has been performed at temperate and boreal latitudes, whereas data from subtropical and tropical inland waters remain scarce^22^. However, the first upscaling approaches have ranked tropical and subtropical systems as major sources of GHG emissions^23^,^24^. In a comparative study of India’s major inland water types, freshwater bodies were shown to emit large amounts of CO_2_ and CH_4_ into the atmosphere that corresponded to 42% of India’s estimated land C sink^25^.

The aim of this study was to analyse and understand the impact of water hyacinths on water-column organic C mineralization and GHG (CO_2_ and CH_4_) emissions from a small, tropical water-harvesting lake in South India. We hypothesized that areas covered by water hyacinths will (1) have higher C mineralization rates and therefore lower O_2_ and higher CH_4_ and CO_2_ concentrations but (2) decreased diffusive CO_2_ and CH_4_ emissions because of the lower gas exchange within the plants compared to that in open water.

## Results

### General lake characteristics

The water temperature of the lake did not drop below 20 °C during three field campaigns in February and November 2012 and March/April 2014 (Table 1), and the lake was slightly alkaline, with a pH between 7.6 and 8.6. In March/April 2014 the mean TIC and TOC concentrations were 7.92 ± 3.24 mmol L^−1^ and 13.5 ± 0.8 mg L^−1^, respectively (Table 1).

**Figure 1:**
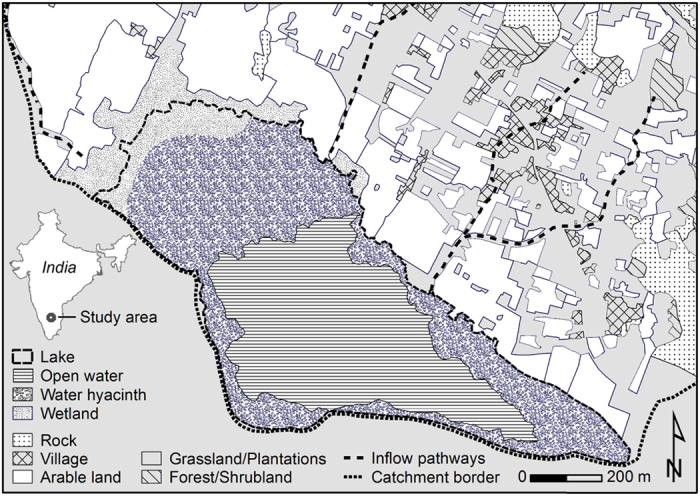
Water hyacinth coverage of Lake Thimmapuram on April, 10th 2014. The extent of the water hyacinth dispersal was determined by GPS from a boat driven along the outer boundaries of the patches. The area of the open water is 0.28 km^2^ (44%); the area of the water under the water hyacinth cover is 0.32 km^2^ (50.4%); and the wetland area is 0.04 km^2^ (5.6%) [ESRI ArcGIS 10.2.1; http://www.esri.com/software/arcgis/arcgis-for-desktop].

**Figure 2:**
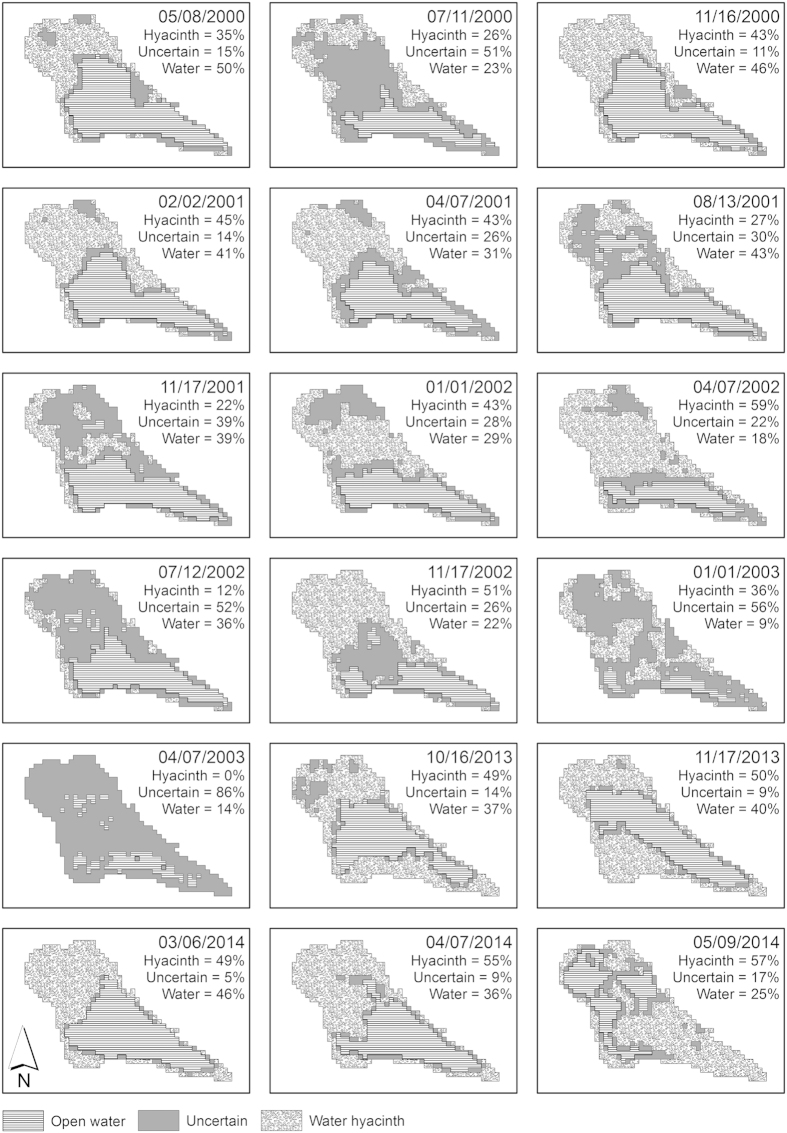
Surface cover classification of Lake Thimmapuram for 2000-2003 and 2013-2014. A threshold-based approach was applied to the Landsat 7 and 8 enhanced vegetation index product, which derived two distinct lake cover classes (water and hyacinths) and an uncertain class because mixed pixels of surface water and water hyacinths could not be separated from the dried lake bottom. The coverage of water, hyacinths and the uncertain areas is provided as the proportion of the total lake area (0.68 km^2^) [ESRI ArcGIS 10.2.1; http://www.esri.com/software/arcgis/arcgis-for-desktop].

The population of water hyacinths on Lake Thimmapuram covered 12 to 55% of the surface area of the lake, with the maximum coverage of 55% reached in April 2014 (Figs. 1 and 2). The dispersal of water hyacinths is strongly managed because the plants are harvested and used as fodder for cattle. Additionally, fishermen occasionally remove the majority of the plants to improve fishing efficiency. In addition to the direct measurements during our field campaign, we derived the coverage of water hyacinth for 2000-2003 and 2013-2014 from remote sensing data (Landsat 7 and 8, Fig. 2). The enhanced vegetation index (EVI) was used for the classification, which introduced a degree of uncertainty, particularly because mixed surface water and water hyacinth pixels are difficult to separate from dried lake bottom pixels (see details in Fig. 2). Water hyacinths could be detected in all available Landsat images, except on April, 4^th^ 2003, when a high percentage of uncertainty was encountered. However, from April, 7^th^ – 10^th^ 2014 (Figs. 1 and 2), the two methods of water hyacinth coverage estimation, on-site GPS recording and remote sensing, produced similar results.

### Water hyacinth-covered areas versus open water

**Figure 3:**
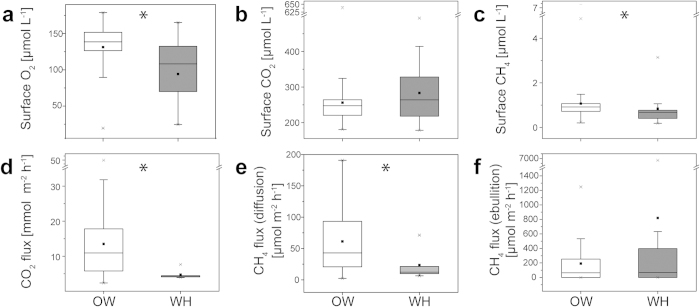
Surface water O_2_(**a**), CO_2_ (**b**), and CH_4_ (**c**) concentrations as well as CO_2_ (**d**) and CH_4_ fluxes as diffusion (**e**) and ebullition (**f**) in the open water (OW) and water hyacinth (WH) areas. Boxplots indicate the medians, the 25th and 75th percentiles (boxes), the 5th and 95th percentiles (whiskers) and the mean values (black squares). Significant differences are denoted with asterisks.

The mean O_2_ concentrations under the water hyacinths (94 ± 46 μmol L^−1^) were lower compared with that of open water (131 ± 37 μmol L^−1^), and the Mann-Whitney *U* test detected a statistically significant (Table 2, p < 0.05) difference between the distributions (Fig. 3a). Average CO_2_ surface concentrations were 283 ± 87 μmol L^−1^ under the hyacinths and 256 ± 77 μmol L^−1^ in open water and were not significantly different between the two zones of the lake (Fig. 3b, Table 2). A significant difference was observed for the distribution of surface CH_4_ concentrations. The mean surface concentrations of CH_4_ were 0.84 ± 0.80 μmol L^−1^ under the hyacinths and 1.07 ± 0.90 μmol L^−1^ in open water (Fig. 3c, Table 2).

**Figure 4:**
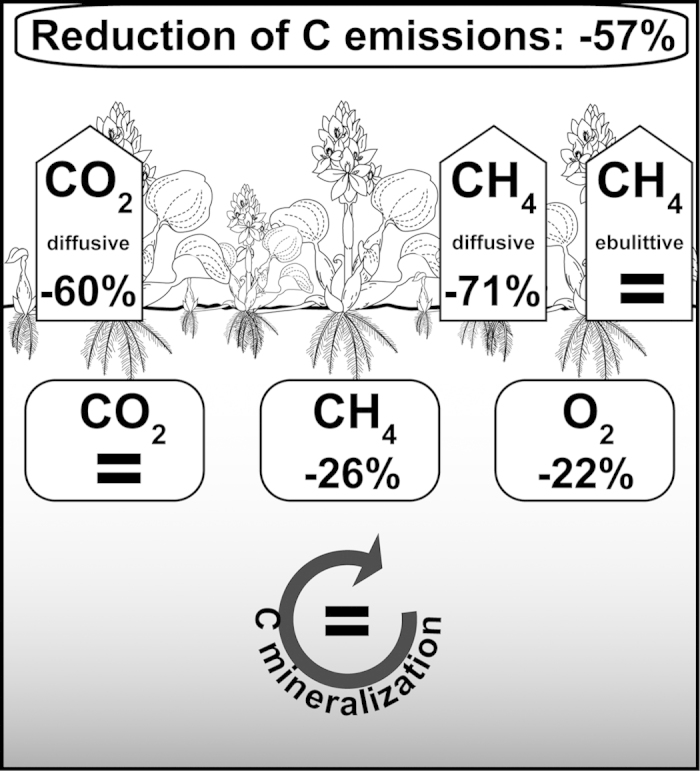
Schematic overview of the major parameters influenced by water hyacinth coverage (CH_4_ and CO_2_ concentrations and their respective fluxes, O_2_ concentrations and carbon (C) mineralization). Significant differences are displayed as the percent reduction of the median fluxes from areas covered by water hyacinths compared with the open water. Non-significant results are denoted by equal signs.

The CO_2_ fluxes from the open water areas were highly variable and ranged from 2.4 to 49.8 mmol m^−2^ h^−1^, with a mean of 13.5 ± 10.2 mmol m^−2^ h^−1^ (Fig. 3d). The CO_2_ fluxes from the lake areas with water hyacinths were less variable and ranged from 3.9 to 7.6 mmol m^−2^ h^−1^, with a mean of 4.7 ± 1.2 mmol m^−2^ h^−1^. The diffusive CH_4_ fluxes were generally lower than the CO_2_ fluxes and ranged from 2.3 to 190.7 μmol m^−2^ h^−1^ in open water and from 6.5 to 71.3 μmol m^−2^ h^−1^ between the hyacinths (Fig. 3e). The diffusive CO_2_ and CH_4_ emissions were significantly higher in open waters than in areas covered by water hyacinths (Table 2, p < 0.05). The distribution of CH_4_ ebullition fluxes, however, was not significantly different between the two areas based on the Mann-Whitney *U* test (range from 0-6,813 μmol m^−2^ h^−1^), although the total C emissions (CO_2_ + CH_4_) from areas covered by water hyacinths were 57% lower than that in open water (Fig. 4).

C mineralization rates in the water column ranged from 102.7 to 526.2 μg C L^−1^ d^−1^ in open water and 138.4 to 599.1 μg C L^−1^ d^−1^ under the water hyacinths, and the Mann-Whitney *U* test did not detect significant (p < 0.05) difference between the distributions of data (data not shown). The O_2_ concentrations at the start of the water incubations from the vegetated areas were lower and reflected the conditions observed directly in the field (158 ± 57 μmol L^−1^ in the water incubations from vegetated areas; 207 ± 29 μmol L^−1^ in the water incubations from open water). Anoxic conditions were not observed in any of the water incubations, and such conditions would have diminished the mineralization rates.

## Discussion

In Lake Thimmapuram, 0.48 to 1.03 million plants per hectare were counted, and their dry weight totalled 16.6 to 35.5 metric tons of dry weight per hectare. The abundance of water hyacinths in Lake Thimmapuram varied strongly between years (from 12 to 55%), although the lake was never completely covered (Figs. 1 and 2), which is presumably because of management by the local villagers and fishermen who depend on the lake for survival. Hyacinth mats can also disperse when there is enough wind, and such a dispersal has also been observed in strongly managed water bodies in the northern part of Bangalore City^9^.

The observed concentrations of O_2_ and CH_4_ in the surface waters of the areas covered by water hyacinths were significantly lower (22% and 26% lower, respectively) than the concentrations in the open water areas, whereas differences were not observed in the concentration of CO_2_ (Figs 3a–c,4). Reduced O_2_ concentrations and even anoxic conditions have also been observed in vegetated areas in other systems covered by water hyacinths^26^ and other floating species^5^,^15^. As we did not measure differences in C mineralization in the water column itself, the reduced O_2_ conditions could be attributed to higher respiration rates at the roots of the plants or in the sediment under water hyacinths. However, in Lake Thimmapuram, the O_2_ content during the day below the vegetated areas was not completely depleted during our sampling campaign, which prevented anaerobic metabolism in the water column and thus affected the C turnover rates, the CO_2_ and CH_4_ concentrations. We did not measure the O_2_ concentrations during the night when the potential for anoxia increases because of an absence of primary production caused by light limitations^27^. However, this potential remains speculative. In addition, CO_2_ and CH_4_ concentrations may also be higher at night.

Surprisingly, the CH_4_ concentrations were lower in the areas covered by water hyacinths, although similar or even higher concentrations might be expected because of the lower O_2_ concentrations and higher organic C content in the sediments. These conditions fuel methanogenesis, as observed in other studies of floating plants^6^,^17^,^28^. The lower surface CH_4_ concentrations beneath the vegetation could be caused by CH_4_ oxidizers living on the roots of the water hyacinths^29^,^30^. For example, Brix and co-authors^31^ found that up to 76% of the CH_4_ produced in the sediment was re-oxidized within the rhizosphere of *Phragmites australis*, which might explain the simultaneously lower concentrations of O_2_ and CH_4_ beneath the water hyacinth because O_2_ is required for the aerobic oxidation of CH_4_.

CO_2_ is an end product of both aerobic and anaerobic respiration^32^. In Lake Thimmapuram, CO_2_ concentrations were not significantly different between the water hyacinth and open water areas, suggesting that the metabolic rates were comparable. This assumption is supported by the similar aquatic C mineralization rates in both areas. A comparison between an area covered by yellow water lilies (*Nuphar lutea*) and an adjacent plant-free zone did not indicate significant differences in the water chemistry^28^, which is consistent with our results for CO_2_. However, the mean CO_2_ concentrations tended to be slightly higher in the surface waters covered by water hyacinths in our study (Fig. 3b). In the central Amazon River and its floodplains, it has been shown that pCO_2_ increased consistently from open water areas towards emergent plants including floating macrophytes^33^ which is consistent with our results. The authors mainly attribute the increases in CO_2_ to an increased supply with organic C from the litter fall and root exudation as well as a release of plant-respired CO_2_ from the roots. However, this is uncoupled from O_2_ consumption in the water column because O_2_ is supplied from the atmosphere. This might explain why we observed different patterns in CO_2_ and O_2_ concentrations.

Furthermore, the differences in CO_2_ concentrations may have been masked by the generally higher CO_2_ concentrations compared with the O_2_ and CH_4_ concentrations (CO_2_ concentrations were 2 and 200-300 times higher than the O_2_ and CH_4_ concentrations, respectively) and a high spatial heterogeneity. Nevertheless, the relatively small but significant differences (O_2_ and CH_4_) or lack (CO_2_) of differences in the concentrations of O_2_, CH_4_ and CO_2_ between the water hyacinth-covered areas and open water might have resulted from the drift dynamics of the water hyacinth mats caused by changing wind directions during the day (personal observation) or lateral mixing of the water body driven by different heating and cooling and densities over the day and night cycle^34^. The drifting was also described by Abdel-Tawwab^35^, who only found a significant decrease in nutrient and O_2_ concentrations and phytoplankton biomass in artificial fish ponds if the free-floating plant (*Azolla pinnata*) cover was greater than 50%, which hinders plant drift.

Generally, the water was supersaturated with CO_2_ and CH_4_ relative to the atmosphere, which led to a net emission of both gases across the air-water interface. By comparing the open water areas and the water hyacinth-covered areas, we found a significant reduction in diffusive C emissions between the covered areas and the open water (Fig. 3d–f,4). CO_2_ emissions could be further diminished in water hyacinth-covered areas because of CO_2_ fixation through photosynthesis^5^,^6^,^28^. However, photosynthetic C fixation by water hyacinths was not quantified in this study. In boreal studies, vegetated littoral areas in aquatic systems have been shown to have the highest areal CH_4_ emissions^36^, which are mostly generated through aerenchymal transport from the emergent macrophytes rooting in sediments that connects the sediment directly to the atmosphere^36^. This mechanism was not relevant for the floating water hyacinths in Lake Thimmapuram, indicating that they must play a different role in the release of GHGs from aquatic systems.

According to our hypothesis, the diffusive emissions of both CO_2_ and CH_4_ were reduced in the areas with water hyacinths. Differences in the surface water concentrations of CO_2_ as a driver of diffusive fluxes can be excluded because differences were not observed in the CO_2_ concentration between the open water and hyacinth-covered areas. Nevertheless, the emitted gases can be trapped inside the plant canopy, which results in a decreased concentration gradient and thus a reduced diffusion. Furthermore, the gas transfer velocity between water and the atmosphere is positively related to the turbulence in the upper water column^37^,^38^,^39^ and the concentration gradient between the media. Water hyacinths reduce the wind speed at the water surface by greatly increasing the roughness length (zone above the surface where the wind speed equals 0 m s^−1^)^40^. Thus, both the concentration gradient between the water and the atmosphere as well as the turbulence of the surface waters were reduced, leading to a reduced exchange of CO_2_ and CH_4_ across the air-water interface among water hyacinths. Similar mechanisms might be expected in other floating-leaved macrophyte communities, such as *Lemna* spp. or *Trapa natans*, which are often found in eutrophic lakes worldwide^4^.

In related studies comparing gas emissions from open water and macrophyte covered areas contradictory results were found. In a study in the Pantanal region, a higher emission of CH_4_ from water hyacinth mats were detected^41^ but other authors^42^ found no differences in the Amazon floodplain between open waters, floating emergent macrophytes, and flooded forests. However, these authors did not determine CO_2_ emissions and the gas fluxes were mainly dominated by CH_4_ ebullition which we do not discuss further here. These different results highlight the demand for further studies to elucidate the role of floating macrophytes for GHG emissions.

We upscaled the CO_2_ and CH_4_ emissions from hourly to daily rates (multiplied by 24) to better compare them to other studies. Our CO_2_ emissions with a mean CO_2_ diffusion rate of 323.8 mmol m^−2^ d^−1^ in the open water and 113.4 mmol m^−2^ d^−1^ among the water hyacinths were well within the range of reported CO_2_ fluxes from aquatic systems in India (from -28.2 mmol m^−2^ d^−1^ 25 to 979 mmol m^−2^ d^−1^ 43). The CO_2_ emissions were approximately 3 times higher and the diffusive CH_4_ fluxes were 2 times lower from open water in Lake Thimmapuram compared with that of the manmade tanks and ponds in India investigated in other studies^25^. Those differences can be directly attributed to physical characteristics, such as turbulence, or indirectly to biogeochemical processes that are influenced by temperature as well as O_2_, C and nutrient concentrations^44^,^45^. Our results highlight the substantial GHG efflux potential of the analyzed lake type (manmade tanks and ponds), which belongs to the major of Tamil Nadu^46^.

Overall, the concentrations of O_2_ and CH_4_ as well as the C emissions from the areas covered by water hyacinths were reduced compared with that of open water. However, the CO_2_ concentrations and water C mineralization rates were not significantly different between the two areas (Fig. 4). These results reveal that invasive water hyacinths can play an important role in biogeochemical processes as well as in the release of climate-relevant gases into the atmosphere. Floating macrophytes, especially invasive species, might therefore be considered as important regulators of gas exchange at the air-water interface, a process that might be central in regional C budgets.

## Methods

### Field campaign and study site description

The water body investigated in this study is Lake Thimmapuram (12.45°N, 78.22°E), which is located in South India (Tamil Nadu State) near the town of Krishnagiri (Fig. 1). The climate is typical of wet and dry tropical regions, with pronounced precipitation seasonality and minor temperature seasonality. The long-term mean annual precipitation in Krishnagiri is approximately 780 mm (measured at the nearby Krishnagiri Dam), and a primary rainy season occurs that is related to the southwest and northeast monsoons between August and November. The mean annual air temperature is 26.4 °C^47^. The lake is eutrophic and shallow (mean depth 1.5 m in March/April 2014) and serves as an irrigation reservoir for the surrounding arable land. The water level in the lake depends on the natural inflow during the monsoon season and the management of a cascade of upstream water-harvesting structures. Following the end of the rainy season, the lake receives additional inflow from December to approximately April via the Krishnagiri Dam (personal communication with dam management). Additional details on the study site can be found in Fiener and co-authors^48^.

The initial sampling campaigns were conducted in 2012 (Table 1), and an intensive sampling campaign was performed in March/April 2014, during which the rates of water-column organic C mineralization and GHG emissions were measured along with the physicochemical water variables (temperature, pH, O_2_, conductivity, total organic carbon (TOC), total inorganic carbon (TIC) and ammonium).

Multi-temporal observations of the lake’s water hyacinth cover were performed by classifying 18 Landsat 7 and 8 scenes (Google Earth Engine) based on the enhanced vegetation index (EVI;^49^) (Fig. 2). Instead of using the more common normalized differenced vegetation index (NDVI;^50^), the EVI was used because of its reduced susceptibility to atmospheric influences and improved sensitivity in high biomass environments^51^,^52^. A simple but robust threshold approach was applied to the EVI product: surface water was classified by an EVI threshold <0.1, and water hyacinths were classified by an EVI >0.3. EVI values between 0.1 and 0.3 were declared to be uncertain because separating a pixel containing both surface water and water hyacinths from the dried lake bottom was impossible. Water hyacinth coverage was also recorded by a Global Positioning System (GPS) from a boat, and the biomass inside a frame (1460 cm^2^; total of six replicated samples on 9 and 11 April 2014) positioned on the water hyacinth meadows was sampled by hand. The plants were washed *in situ*, separated into emerged and submersed leaves plus roots, and desiccated at 70 °C until they reached a constant weight.

### Physicochemical variables

O_2_, pH, conductivity (corrected to 25 °C), and temperature were measured with a YSI probe (YSI Inc., Yellow Springs, OH, USA). Gas samples for the analysis of dissolved CO_2_ and CH_4_ were obtained using the headspace extraction technique^53^. Water samples (20 mL) were collected from the surface waters (~10 cm depth) in glass vials equipped with septa, and the vials were immediately closed and kept gastight without a headspace. Subsequently, a 5 mL headspace was created with ambient air, the vials were vigorously shaken for 60 seconds, and 500 μL gas samples were then collected from the headspace with a gastight syringe and manually injected into a closed loop between the gas inlet and the outlet of a Los Gatos GHG analyzer (Los Gatos Research Inc., Mountain View, CA, USA) to measure the CO_2_ and CH_4_ contents^54^. This method, first described by Baird and co-authors^55^, allows a fast on-site determination of CO_2_ and CH_4_ gas samples. The volume of the loop was 72.6 ± 2.2 mL and precision of measurements amounted to 3-5%. The samples used for the analysis of total inorganic carbon (TIC) were prepared following the same procedure as the CO_2_ samples but with the addition of phosphoric acid (pH < 4) before shaking to outgas the inorganic carbonate species as CO_2_. The partial pressures of the gases were converted into concentrations in water (expressed as μmol L^−1^) by using Henry’s constant, the water temperature, and the measured gas partial pressures in the air (while accounting for the water volume and the headspace inside the bottle)^56^. Overall, 139 surface samples were collected at random locations across the lake over 13 days during the March/April 2014 sampling campaign and used to measure the concentrations of CO_2_ and CH_4_. The analysis of the TOC from the surface waters (~10 cm depth) was performed using a TOC analyzer (Shimadzu Co., Kyoto, Japan) according to method 5310^57^.

### Greenhouse gas emissions

The GHG flux (CO_2_ and CH_4_) across the water-atmosphere interface was measured with floating chambers that were gently deployed from a boat onto the water surface between water hyacinths and in open water areas to minimize artificial turbulence. Similar to the protocol described in McGinnis and co-authors^39^, the chambers were constructed of inverted non-transparent plastic buckets with a volume of 14.76 L and an area of 1,018 cm^2^. Some light could have penetrated through the plastic, however, this should not have changed the GHG emissions on these short timescales (20 min). A floating device composed of polyethylene was attached to the chambers, and approximately 2 cm of the chamber walls was allowed to submerge to ensure a gastight seal between the water surface and the chamber while minimizing the impact of the natural turbulence in the water column beneath the chamber^58^. Two gas ports (inlet and outlet) were fitted on top of each chamber and connected with 2 × 5 m-long gastight tubes (Tygon 2375) to a Los Gatos ultraportable GHG analyser. The internal pump circulated the air in the gas chamber through the GHG analyser at a rate of ~450 mL min^−1^. The boat and the chambers were allowed to drift freely on the lake surface for 10-20 min per deployment, and the concentrations of CO_2_ and CH_4_ were measured every second, which allowed the changes in CO_2_/CH_4_ to be tracked *in situ*. The concentrations of CH_4_ and CO_2_ inside the atmosphere of the chamber increased linearly over time under diffusional conditions, whereas the CH_4_ concentrations increased abruptly when bubbling occurred. This process allowed us to separate the bubbling and the strict diffusional flux by the high sampling frequency enabled by the GHG analyser^59^. However, the short incubation time did not allow an accurate determination of CH_4_ ebullition and is thus not further emphasized in the discussion. The water-atmosphere fluxes (*J*) of CO_2_ and CH_4_ (mmol m^−2^ h^−1^ and μmol m^−2^ h^−1^, respectively) were calculated from the slopes (*s*) of the linear regressions of the concentrations in the chamber versus time as follows:


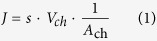


where *V*_ch_ is the chamber volume, and *A*_ch_ is the chamber area. The amount of gas released per bubbling event was determined by calculating a two-point regression from the concentrations in the chamber at the start of the bubbling event and after the bubbling event, when the CH_4_ concentration in the chamber was well-mixed^59^. Fluxes were only measured during the day because local circumstances did not allow for night measurements. In total, 41 chamber measurements were performed at different locations on eight different days during the three-week sampling campaign.

### Carbon mineralization

Water column C mineralization was determined using transparent acryl-glass incubation cores (length of 30 cm and inner diameter of 5.4 cm) that contained a septum in the tube wall for *in situ* O_2_ measurements. The incubation containers were carefully filled with water collected at the water surface in the vegetated and open areas. We avoided collecting any plant remnants during the filling in the vegetated areas, which would have increased our mineralization rates. After applying an airtight seal to the containers, respiration was quantified for the water samples by O_2_ depletion over 24 hours. The incubation cores were incubated at *in situ* temperatures in the dark. O_2_ depletion was measured with a needle-type O_2_ microsensor (Optode, PreSens, Regensburg, Germany) after the water column was mixed, and the amount of consumed O_2_ was converted to μg C L^−1^ d^−1^ using a conversion factor of one^60^. A more detailed description is given in Attermeyer and co-authors^61^.

### Statistics

Because normal distributions were not observed for all of the parameters, we tested for differences in the chemical variables, CO_2_ and CH_4_ emissions, and water C mineralization under the water hyacinths and in open water using a non-parametric, two-sided Mann-Whitney *U* test 62. To consider the temporal differences during the sampling periods, all of the values of each group (water hyacinths and open water) from different days of the sampling campaign in March/April 2014 were included. Differences in the distribution of the different groups were considered significant at p < 0.05. All of the values were expressed as the mean ± standard deviation, and all of the statistical analyses were performed with IBM SPSS Statistics 22 (IBM Corporation, Armonk, NY, USA).

## Additional Information

**How to cite this article**: Attermeyer, K. *et al.* Invasive floating macrophytes reduce greenhouse gas emissions from a small tropical lake. *Sci. Rep.* **6**, 20424; doi: 10.1038/srep20424 (2016).

## Figures and Tables

**Table 1:** Water physicochemical variables from several sampling stations on Lake Thimmapuram summarized from sampling campaigns conducted in February and November 2012 and March and April 2014.

	February 2012			November 2012			March/April 2014		
Parameter	Mean ± SD	Min	Max	Mean ± SD	Min	Max	Mean ± SD	Min	Max
Temperature [°C]	25.6 ± 1.6	24.1	27.3	22.0 ± 0.2	21.7	22.3	29.1 ± 0.9	27.8	30.6
n*		*6*				*8*		*10*	
pH	8.1 ± 0.1	8.0	8.2	8.3 ± 0.2	7.9	8.6	7.8 ± 0.1	7.6	8.0
*n*		*6*				*8*		*10*	
Conductivity [μS cm^-1^]	1346 ± 3	1343	1350	1182 ± 42	1101	1222	1553 ± 29	1501	1595
*n*		*6*			*7*			*10*	
O_2_ [mg L^-1^]	11.0 ± 3.3	8.1	16.8	0.41	nd	nd	3.65 ± 1.27	1.86	5.22
*n*		*6*			*1*			*10*	
TIC [mmol L^-1^]	nd	nd	nd	nd	nd	nd	7.92 ± 3.24	7.34	8.33
*n*								*10*	
TOC [mg L^-1^]	nd	nd	nd	nd	nd	nd	13.5 ± 0.8	12.4	14.9
*n*		*9*			*9*			*10*	
Water depth [m]	1.3 ± 0.6	0.4	2.3	0.7 ± 0.4	0.2	1.5	1.5 ± 0.5	0.7	3.1
*n*		*9*			*9*			*36*	

For a better comparability, only open water samples are compiled in the table. ^*^n is sample size.

**Table 2:** Statistics for the comparison between the open water area and water hyacinth-covered area.

	Open water			Stands of water hyacinths			Mann Whitney *U*		
Parameter	Mean ± SD	Min	Max	Mean ± SD		Min	Max	U	p
O_2_ conc [μmol L^-1^]	131 ± 37	20	179	94 ± 46		25	165	1124.5	**0.001**
n*		*87*			*17*				
CO_2_ conc [μmol L^-1^]	256 ± 77	180	642	283 ± 87		178	494	972	0.191
*n*		*119*			*20*				
CH_4_ conc [μmol L^-1^]	1.07 ± 0.90	0.20	7.42	0.84 ± 0.80		0.19	3.14	1687	**0.003**
*n*		*119*			*20*				
CO_2_ em (diffusive) [mmol m^-2^ d^-1^]	13.5 ± 10.2	2.4	49.8	4.7 ± 1.2		3.9	7.6	50	**0.001**
*n*		*31*			*10*				
CH_4_ em (diffusive) [μmol m^-2^ h^-1^]	61.2 ± 45.8	2.3	190.7	23.5 ± 22.7		6.5	71.3	75	**0.004**
*n*		*42*			*9*				
CH_4_ em (ebullitive) [μmol m^-2^ h^-1^]	191 ± 294	0	1248	819 ± 2116		0	6814	150	0.887
*n*		*29*			*10*				
Water C min [μg C L^-1^ d^-1^]	326 ± 120	103	526	389 ± 146		138	599	174	0.166
*n*		*22*			*21*				

Statistically significant p-values by the Mann Whitney U test are displayed in bold. ^*^n is sample size
